# Electrospun nerve guide conduits have the potential to bridge peripheral nerve injuries in vivo

**DOI:** 10.1038/s41598-018-34699-8

**Published:** 2018-11-13

**Authors:** Hanna K. Frost, Tomas Andersson, Sebastian Johansson, U. Englund-Johansson, Per Ekström, Lars B. Dahlin, Fredrik Johansson

**Affiliations:** 1 Department of Translational Medicine – Hand Surgery, Lund University, SE-205 02 Malmö, Sweden; 2 Department of Hand Surgery, Skåne University Hospital, SE-205 02 Malmö, Sweden; 3 Department of Biology, Lund University, SE-223 62 Lund, Sweden; 4 Department of Clinical Sciences in Lund - Ophtalmology, Lund University, SE-211 84 Lund, Sweden

**Keywords:** Regeneration and repair in the nervous system, Implants

## Abstract

Electrospinning can be used to mimic the architecture of an acellular nerve graft, combining microfibers for guidance, and pores for cellular infiltration. We made electrospun nerve guides, from polycaprolactone (PCL) or poly-L-lactic acid (PLLA), with aligned fibers along the insides of the channels and random fibers around them. We bridged a 10 mm rat sciatic nerve defect with the guides, and, in selected groups, added a cell transplant derived from autologous stromal vascular fraction (SVF). For control, we compared to hollow silicone tubes; or autologous nerve grafts. PCL nerve guides had a high degree of autotomy (8/43 rats), a negative indicator with respect to future usefulness, while PLLA supported axonal regeneration, but did not outperform autologous nerve grafts. Transplanted cells survived in the PLLA nerve guides, but axonal regeneration was not enhanced as compared to nerve guides alone. The inflammatory response was partially enhanced by the transplanted cells in PLLA nerve grafts; Schwann cells were poorly distributed compared to nerve guide without cells. Tailor-made electrospun nerve guides support axonal regeneration *in vivo*, and can act as vehicles for co-transplanted cells. Our results motivate further studies exploring novel nerve guides and the effect of stromal cell-derived factors on nerve generation.

## Introduction

In spite of intense research, peripheral nerve injuries still constitute a challenging clinical problem. Surgical repair is the only established treatment of complex, extensive peripheral nerve injuries in the upper extremity, but often results in suboptimal functional recovery with loss of sensation and paresis^1,2^. Autologous nerve grafting of a less important nerve is the current gold standard for reconstruction of a nerve injuries with a defect. However, autologous nerve grafts are limited by issues related to availability, since unimportant nerves must be used in parallel bundles to match the diameter of the injured nerve trunks.

In spite of different germ layer origins, adipose derived stem cells have been speculated to improve regenerative outcomes after a peripheral nerve injury^3,4^. Early surgical nerve repair and reconstruction of injuries is favorable compared to delayed procedures^5,6^, stressing the need for immediately available cell sources for any therapeutic intervention. Recent studies have examined the addition of uncultured stromal vascular fraction (SVF) to hollow tubes and vessel grafts in attempts to improve peripheral nerve regeneration in rats^7–10^. The SVF is obtained after enzymatic digestion of adipose tissue and elimination of floating mature adipocytes, and contains, among many other cell types, a fraction of adipose-derived stromal and stem cells (ASC)^11^. Stem cell yield is higher from adipose tissue than bone marrow, and one gram of aspirated adipose tissue yields approximately 3.5 × 10^5^ to 1 × 10^6^ ASCs^12^ - a rich and convenient source of cells.

One of the challenges with somatic cell therapy is how to dock the transplanted cells to the intended area of action, and avoiding ectopic engraftment^13^. *In vitro* studies have suggested that nanofibrous aligned poly-L-lactic acid (PLLA) scaffolds may function as potential stem cell carriers to injured nerve sites^14^. Further, aligned nanofibers are known to provide topical guidance and enhance neurite outgrowth speed and orientation in dorsal root ganglia explants^15–17^. Nanofibers may thus play a dual role for topical guidance as well as cell adhesion of transplanted cells.

Embracing these demands, electrospinning can be used to create fibers and 3D structures from biodegradable and biocompatible polymers, with an appealing range of possible applications in regenerative medicine^18,19^.

The purpose of this study was to combine the two approaches of a well-tailored bioengineered nerve conduit and cell therapy using SVF in an *in vivo* setting. We manufactured nerve guides with micro channels, whose walls are lined with parallel fibers for topical guidance. Using two polymer materials, polycaprolactone (PCL) and PLLA, we investigated the *in vivo* performance up to 28 days in a rat model of sciatic nerve injury and reconstruction, and it’s potential to bridge a 10 mm nerve gap, and act as a cell delivery vehicle.

## Results

### Tube characteristics of PCL nerve guide

**Figure 1:**
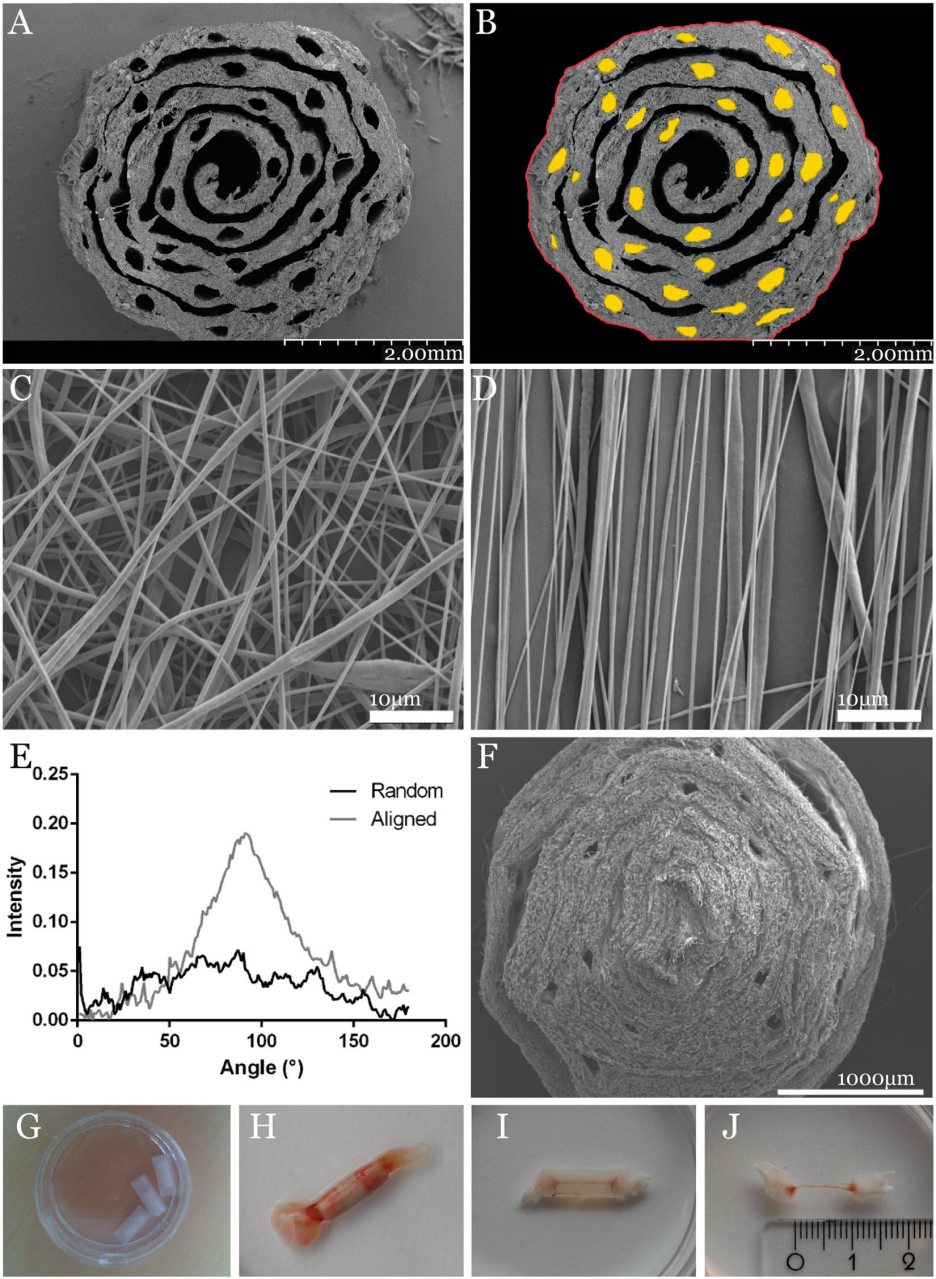
PLC nerve guide characteristics. (**A**) SEM images of the PCL guide with more defined number of micro channels; (**B**) individual channel dimensions traced in ImageJ to calculate the porosity; (**C**) randomly oriented PLLA fibers; and (**D**) aligned PLLA fibers, indicating the porosity of the scaffold, and (**E**) the Fourier spectra of the random and aligned fibers. (**F**) SEM image of the PLLA nerve guide, where the pores appear to be less well defined than in the PCL guide; (**G**) the PCL nerve guide inserted in its silicone shell; (**H**) explanted nerve guide integrated in the regenerating sciatic nerve; (**I**) hollow tube bridged by a thin silicone matrix; (**J**) the regenerated matrix measured to the length of the sciatic nerve defect of 10 mm.

The majority of channels in the guides (in average 27 channels) were still intact after fabrication. The porosity, i.e. the fraction of unoccupied space in the guides, was approximately 22% of the total area (excluding the central canal); 21% of this area was micro channels with aligned fiber walls. Scanning electron microscopy of aligned (Fig. 1C) and random oriented fibers (Fig. 1D), as well as fourier spectra of both fiber types (Fig. 1E) confirmed the intended fiber orientation pattern of the electrospun material. Observation in a scanning electron microscope showed that the PCL- and PLLA nerve guides had micro channels where the sutures had been placed (Fig. 1A,B,F). The micro channels of the PLLA guides were less well defined than in the PCL nerve guides (Fig. 1A,F).

### Animal interruption outcomes

**Figure 2:**
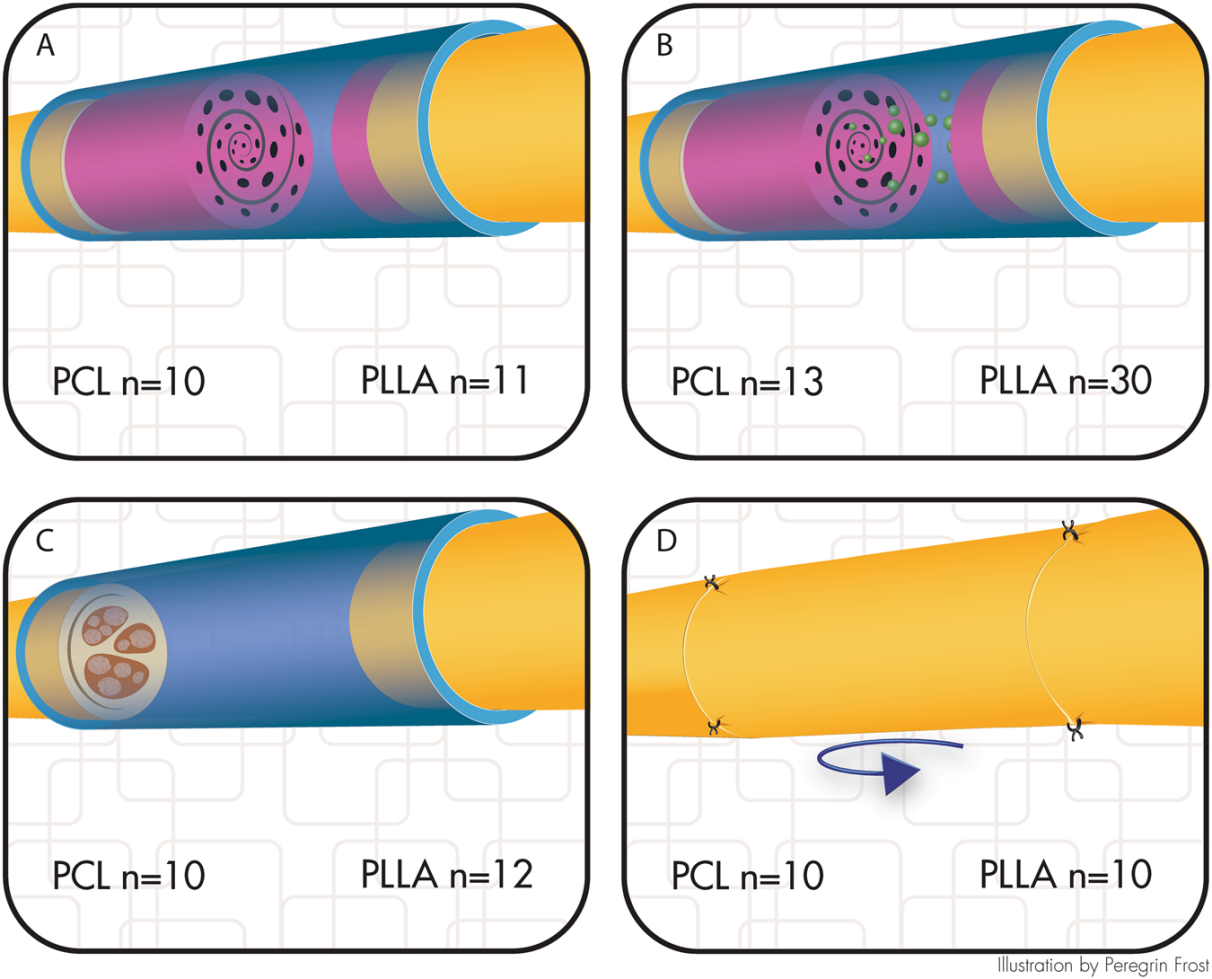
Distribution of animals into four groups. (**A**) nerve guide; (**B**) nerve guide with autologous cell transplant from stromal vascular fraction; (**C**) hollow silicone tube, or (**D**) autologous nerve graft. PCL denotes the part of the study in which PCL guides were used, while PLLA denotes the part of the study where PLLA guides were used, even though such guides where not used when it came to the respective autologous nerve graft and hollow tube groups. (Illustration by Peregrin Frost).

Animal interruption was used as criteria for studying biocompatibility of the two different polymers used. Figure 2 shows the division of animals into four groups.

#### PCL study

We observed a very high degree of autotomy mainly at the implant side, resulting in irreparable skin defects during the first 24 h post-surgery. In the two PCL nerve guide groups, 8/23 rats were terminated because of such autotomy. Corresponding frequencies were 2/10, and 1/10, for hollow tube and autograft, respectively.

#### PLLA study

In the group with SVF cells, 4/30 were terminated because of autotomy at the abdominal incision site, and additional 4/30 were terminated for wound complications after the first week. In the group with nerve guide without cells, 1/11 was terminated at day 22 due to wound on the operated foot. In the group treated with a hollow tube, 1/12 was terminated at day 8 because of a wound at the operated limb. In the autologous nerve graft group, all animals were well and completed the study.

### Macroscopic evaluation after 4 weeks

#### PCL study

In the group with PCL nerve guide w/o cells, 8/10 animals completed the study; four had integrated the fiber nerve guide in the nerve defect; one was discontinuous, and one was found correctly positioned but the proximal nerve end had not grown into the nerve guide. In the group with PCL nerve conduit with SVF cells, 7/13 completed the study; two had integrated the fiber nerve guide in the nerve defect. In one, the construct was discontinuous proximally and the nerve had not grown into the conduit; in three, the nerve conduit had translocated inside the silicone tube in proximal or distal direction, but was continuously bridged (with a thin matrix where the nerve guide had slided away); in addition, some nerve guides had moved prior to implantation and were not used). One appeared to be infected or inflamed and was grossly encapsulated. In the group with hollow tube, 8/10 completed the study; five were macroscopically bridged with a thin regenerative matrix in the silicone tubes; three were discontinuous. In the autologous nerve graft group, 6/10 animals completed the experiments, and their sciatic nerve reconstructions were observed to be normal.

#### PLLA study

In the group with PLLA nerve guide without cells, 10/11 rats completed the experiment; in 8/11 rats, the nerve guides were found correctly positioned and integrated in the sciatic nerve; 1/11 nerve guides had moved proximally inside the silicone shell and the nerve was not bridged. In the group with PLLA nerve conduit with SVF cells, 20/30 rats completed the study; 8/30 were well integrated; 1/30 was integrated proximally but discontinuous distally. Notably, 9/30 appeared to be infected or inflamed, and were encapsulated; some of these nerves were continuous with the nerve guides, and some were not. In the group with hollow tube, 11/12 rats completed the experiment, and a bridging regenerative matrix was formed in all silicone tubes. In the autologous nerve graft group, 10/10 rats completed the study and all nerves were bridged; however, one specimen was injured during harvest and could not be analyzed.

### Cellular distribution

#### PCL study

**Figure 3:**
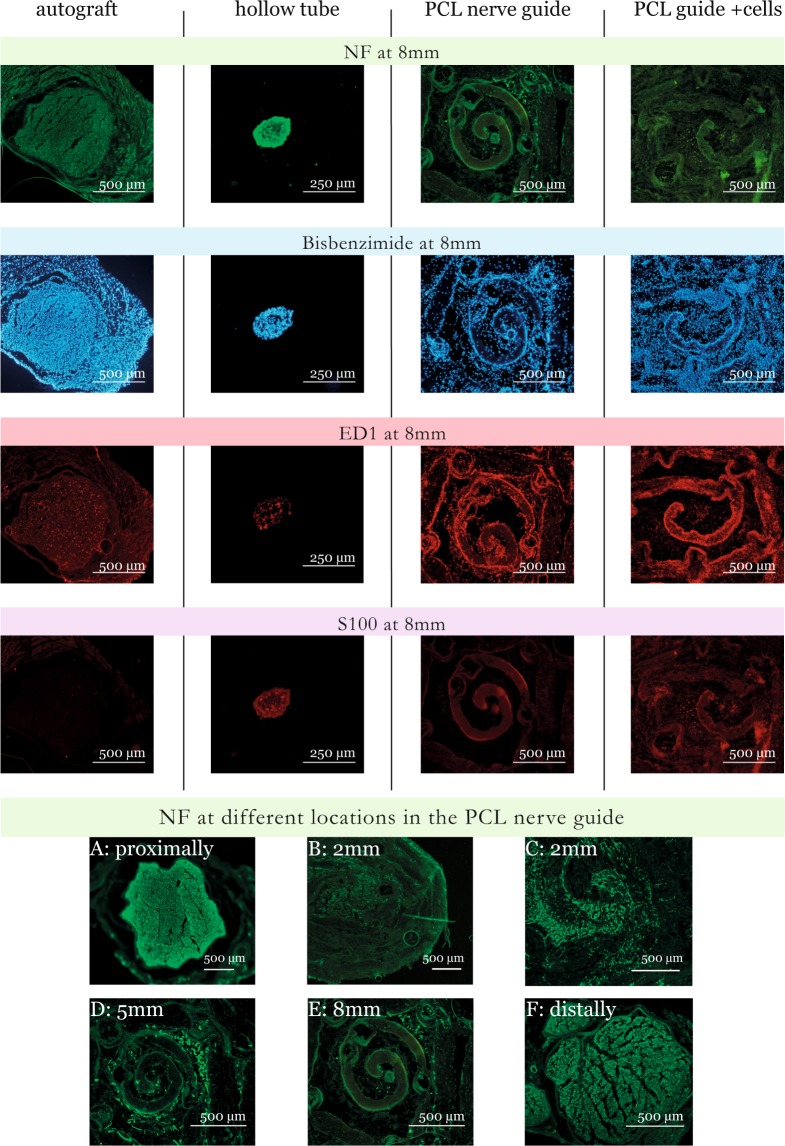
Cross sections from the PCL nerve guide study. Sections were stained for neurofilaments (NF), cell nuclei (Hoechst 33342), macrophages (ED1), and Schwann cells (S-100). It was technically impossible to match all images. Immunofluorescence microscopy rendered a faint background, which is due to light scattering in the fiber matrix and not due to autofluorescence. Newly formed axons were observed in some of the channels (**D**,**E**) but the majority of neurofilaments were found in the space between the fiber mat layers (**D**).

**Figure 4:**
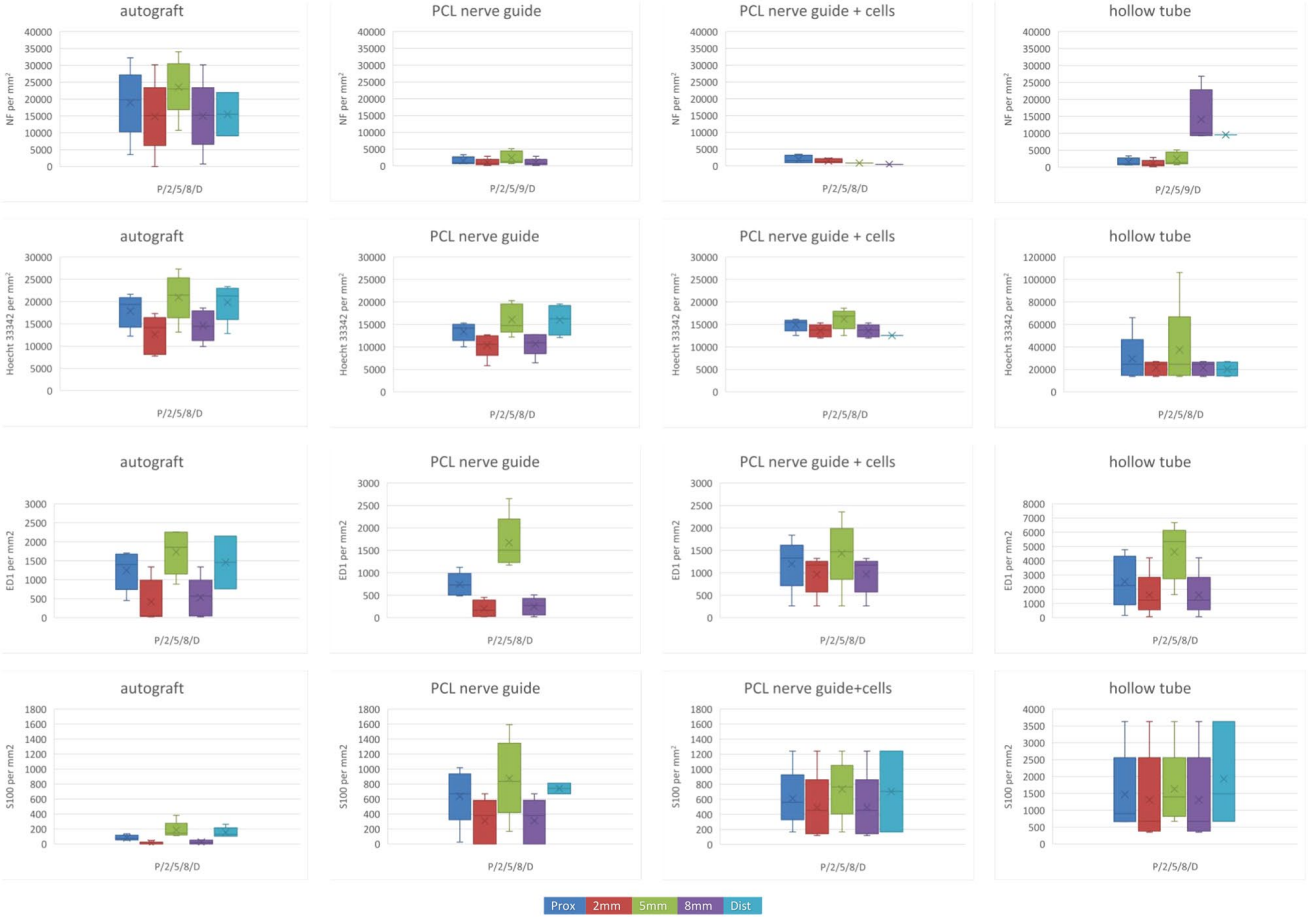
Data from the PCL nerve guide study visualized by boxplots. Boxes indicate median values, 25- and 75 percentiles; and whiskers indicate minimum and maximum values. (**A**) NF stainable structures; (**B**) Hoechst 33342 stained nuclei; (**C**) ED1 stainable structures; (**D**) S-100 stainable structures; all determined by counting at photographs of cross sections taken proximally, at 2,5,8 mm from the site of the proximal suture, and distal to the lesion. To avoid cluttering, the detailed data are found in the Supplemented Table S1. Hollow tube (right panel) is presented with a different scale, and some observations in this group are based on a single specimen. Note that there are less S-100 stainable structures at 8 mm in autograft group than in the PCL nerve guide.

Using Hoechst 33342 (a DNA binding dye) as a nucleus indicator, the total cell count per mm^2^ at cross sections taken in different parts of the nerve guide is presented in Fig. 3 (blue panel) and Fig. 4. To avoid cluttering, detailed data is presented in the Supplemented Table S1. There was a difference in the cell counts between treatment groups at 5 mm and 8 mm, where the autologous nerve graft had more cell nuclei than in the PCL nerve guide at 5 mm and at 8 mm (KW, all p-values < 0.05). Proximally, at 2 mm or at the distal nerve segment, no differences between groups could be detected.

### Axonal regeneration - neurofilament staining

Neurofilament protein is a component of the neuronal cytoskeleton, and is closely associated with the regeneration of injured axons.

#### PCL study

Newly formed neurofilaments were observed in clusters in defined sections of the PCL guides, primarily close to their center, regardless if cell therapy was used or not (Fig. 3, green panel). The newly formed axons did utilize some of the channels (Fig. 3D,E), but many channels were void of neurofilament stainable structures. Instead, a majority of neurofilaments were found in the space between the fiber mat layers (Fig. 3D). The axons detected inside the channels were observed to be comparably round and symmetrical (not shown), indicating that they grew along the direction of the channels inside the guide. In the channels, the axons displayed a morphology very similar to that observed in the distal nerve segment and in the autologous nerve graft sections. Outside the channels, newly formed axons tended to have an irregular shape, interpreted as a sign of random, multidirectional axonal growth inside the porous matrix, and not perpendicular to the transverse sections of the guides. Occasionally, random growth could also be seen inside (probably damaged) channels, and cross-sections that are more circular were sometimes found between fiber mat layers. In a few specimens, the majority of the axonal growth occurred around the other edges of the guides, and along the inner wall of the silicone shell. Two PCL guides showed no signs of neurofilament positivity at all inside any of the randomly selected transverse sections; these were discarded from quantitative analysis. Both autologous nerve grafts and empty tubes had neurofilaments bridging the 10 mm defect.

The amount of neurofilament stainable structures at cross sections is presented in Table S1, Figs 3 and 4. The groups were small, and no differences between groups could be detected at any position (Table S1).

#### PLLA study

**Figure 5:**
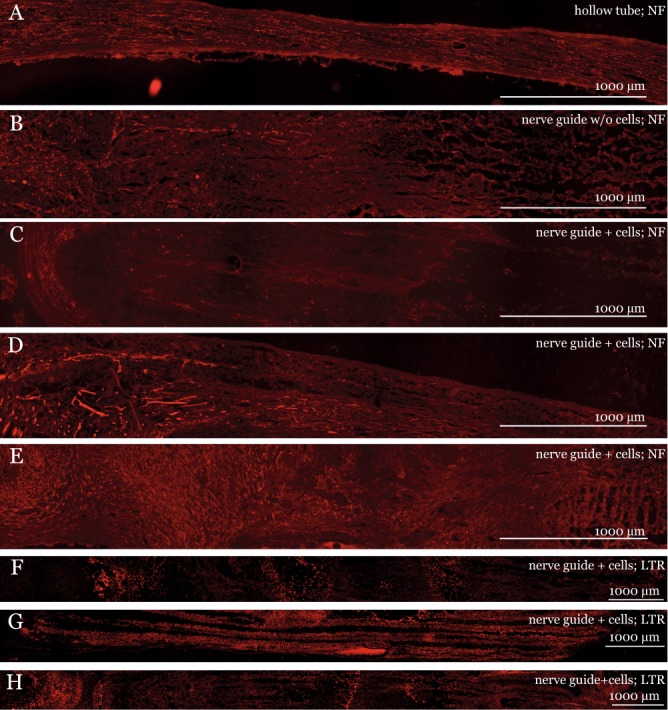
Longitudinal sections of the PLLA nerve guide four weeks after sciatic injury and repair. Panel (A–E): Neurofilament (NF), ED1, and S-100 staining of (**A**) regeneration matrix in a hollow silicone tube; (**B**) nerve guide without cells; (**C**–**E**) and nerve guide with cells. Panel F–H: LipidTOX Red (LTR) staining of PLLA nerve guide + cells. Immunofluorescence microscopy rendered a faint background for both polymer types, which is due to light scattering in the fiber matrix and not due to autofluorescence.

Data is presented in Table 1 and Fig. 5. Axonal outgrowth in autologous nerve grafts and hollow tubes outperformed the PLLA guides with and without cells (KW p < 0.001). Cell therapy with SVF-cells did not significantly alter the axonal outgrowth compared to PLLA nerve guides alone, but there was a trend of inferior axonal outgrowth in the cell therapy group compared to nerve guide alone (MW p = 0.074 and p = 0.059 for maximal and average NF length, respectively). We observed no difference in regenerative length between autologous nerve graft and hollow tube. As with the PCL guide, neurofilament stainable filaments were observed to follow the micro channels (Fig. 5). For the hollow tube, the regenerated matrix was only about half the width of uninjured control nerve, while the graft with the integrated PLLA guide was more than twice the diameter of the autologous nerve graft.

### ED1 staining

ED1 is a marker of activated macrophages and inflammation.

#### PCL study

Data is presented in Table S1 and Figs 3 and 4. The groups were very small, and no differences could be detected between groups at any position (Table S1).

#### PLLA study

Data is presented in Table 1. Proximal to the lesion, more ED1 stainable cells were detected in the PLLA nerve guide + cells compared to autologous nerve graft (KW p = 0.038; MW p = 0.002). Five mm distally, there were less ED stainable cells in the nerve guide compared to autologous nerve graft, and nerve guide + cells had more ED1 stainable cells compared to both autologous nerve graft and compared to nerve guide alone (KW p = 0.007).

**Table 1:** Nerve regeneration after nerve injury and reconstruction with PLLA nerve guide.

	Autologous nerve graft	Hollow tube	PLLA nerve guide	Nerve guide + cells	p-values (KW)
NF max length (mm)	10.00 (8.98, 10.00)	10.00 (6.04, 10.00)^cd^	4.14 (1.46, 9.02)^ab^	3.11 (2.19, 3.94)^ab^	**<0.001**
NF average length (mm)	10.00 (8.98, 10.00)	10.00 (6.04, 10.00)^cd^	3.27 (1.16, 5.61)^ab^	2.59 (1.88, 3.75)^ab^	**<0.001**
ED1 prox (cells/mm^2^)	44 (20, 68)		40 (12, 100)	69 (54, 252)^a^	**0.038**
ED1 5 mm dist (cells/mm^2^)	44 (26, 106)		34 (10, 60)^ad^	88 (6, 194)^ac^	**0.007**
S-100 (length in mm)	9.988 (5.753, 1.184)	9.253 (4.851, 15.142)^d^	9.836 (3.925, 12.865)^d^	3.905 (2.012, 8.906)^abc^	**0.006**

NF, ED1 and S-100 staining of longitudinal sections of the PLLA nerve guide. Values are expressed as median (min, max). KW = Kruskal-Wallis. Statistical significance (p < 0.05) based on the Mann-Whitney U-test is indicated as follows: ^a^significant difference from autologous nerve graft; ^b^significant difference from hollow tube; ^c^significant difference from PLLA nerve guide; ^d^significant difference from PLLA nerve guide + cells.

### S-100 staining

S-100 is used as a marker for Schwann cells, the mail glial cells of the peripheral nervous system. Schwann cells enable and support regeneration in the injured peripheral nerve.

#### PCL study

Data is presented in Table S1, Figs 3 and 4. The groups were very small, but the autograft had less S-100 stainable cells compared to the PCL nerve guide at 8 mm (KW p < 0.05). No differences could be detected between groups at any other position.

#### PLLA study

Data is presented in Table 1. S-100 stainable cells could be located further distally from the site of lesion in all three groups compared to the group treated with PLLA nerve guide + cells (KW p = 0.006).

### LipidTOX Red staining

LipidTOX Red stains neutral lipid droplets present in mature adipocytes. The SVF cell population contains, among other cells, adipocyte precursors.

#### PCL study

The specimen was not analyzed for this feature, since the PCL nerve guide was poorly tolerated and groups were very small.

#### PLLA study

Observation of LipidTOX Red stain, used to visualize transplanted cells from adipose tissue-derived stromal vascular fraction, showed that there were stained cells spread throughout the guides. Furthermore, at the distal end of the guide, there was an accumulation of LipidTOX Red stained cells (Fig. 5G,H).

## Discussion

We investigated the *in vivo* performance of electrospun multi-channeled guides bridging a 10 mm sciatic nerve gap in a rat model of peripheral nerve injury and reconstruction, and examined the possibility to use the implant as a vehicle for cell therapy. We compared the outcome to hollow silicone tubes, previously used to bridge short gaps clinically^20–22^, and to the current gold standard – autologous nerve graft^23^ – a therapeutic approach which cannot always be utilized in large injuries due to a shortage of donor tissue. We found that PCL nerve guides had poor biocompatibility, but that electrospun PLLA nerve guides had better mechanical properties, supported axonal regeneration *in vivo*, and can act as a vehicle for co-transplanted cells up to 28 days.

A thorough search of the relevant literature yielded no other *in vivo* studies of graftable multi-channel nerve conduits, while the use of polymer materials as nerve scaffolds has been previously explored^24,25^. With the design featuring multiple channels, prior reports have focused on the manufacture process and *in vitro* observations. Jeffries *et al*. reported on a nerve conduit with longitudinally-aligned microfibers and pores, mimicking the architecture of the acellular nerve graft and evaluated *in vitro*^26^. Kim *et al*. fabricated nerve conduits with randomly-oriented nanofibers to strengthen the weak mechanical strength of aligned nanofibers used for nerve cell attachment and proliferation guidance^27^. Our electrospun nerve guides did not obtain such a high structural similarity to the acellular nerve grafts as did the electrospun PCL guides by Jeffries and Wang, who report a higher number of channels over a thinner diameter compared to our 3 mm thick guides^26^. An option for improvement could be a better match of the diameter of the nerve guide to the target organ.

We tested two FDA-approved polymers: PCL and PLLA. However, the PCL conduit was poorly tolerated with a high degree of autotomy as previously reported^28^. Electrospun PCL shells have been reported to create a massive foreign body reaction^29^, and fistulation of the commercially available PCL-guide Neurolac™ has been described in humans^30^. In our study, the PCL nanofiber scaffolds were perceived as difficult to handle, and the nerve guides had to be tied together to not unfold. This rendered the PCL guides less attractive, and further studies will focus on PLLA.

In order to obtain external stability to withstand surgical handling during the nerve reconstruction procedure, the nerve guides were inserted in a standard model, i.e. a hollow silicone tube^21,22^. The design with an outer shell, however, turned out to be a weak point, as several nerve guides were discovered to have been moving within the silicone shell at the end of the study period of 28 days.

The mechanical properties of the PCL nerve guide made analysis problematic, as the explanted structure was very difficult to cut in longitudinal sections with the cryotome. PLLA has a higher Young’s modulus than PCL, making it stiffer, and hence the PLLA guides keep their structural integrity. The PLLA nerve guide could better withstand the mechanical forces and was possible to cut in longitudinal sections. Immunofluorescence microscopy rendered a faint background for both polymer types, which is due to light scattering in the fiber matrix and not due to autofluorescence.

As expected, the autologous nerve graft was fully bridged by neurofilaments. Furthermore, a 10 mm nerve defect was reconstructed with a hollow silicone tube, showed newly regenerated axons at four weeks in consistence with previous studies^31^. In hollow tubes of various materials, it is crucial that a fibrin matrix is formed, facilitated by the tube to a certain extent, between the proximal and distal nerve ends inside the tube^32–34^. If the distance between the nerve ends is too long, no matrix is formed, hindering the regeneration process across a nerve defect^35–39^. This is observed also clinically and is considered in clinical practice concerning various conduits and other materials, such as nerve allografts^40^. The fibrin matrix contains relevant cells, such as inflammatory cells (e.g. macrophages), and its formation is a prerequisite for the nerve regeneration process, including migration/proliferation of Schwann cells and axonal growth.

Regenerative axonal length at 28 days, as evaluated by neurofilament staining, was inferior in the electrospun PLLA nerve guide compared to the autologous nerve grafts and the hollow tubes. The structure and porosity of the fiber matrix increase the surface length and area, and the newly regenerated axons extended in different planes inside the confined space compared to the hollow tubes. If a longer gap than 10 mm had been evaluated, the outcome of the nerve guides versus hollow tubes could possibly have been different; further studies could address this issue, as should functional tests be included to evaluate the overall performance beyond the early cellular events at the graft region at 28 days.

The structure appears to allow for infiltration of Schwann cells, the most important cell type for creating a favorable axonal environment and supporting regeneration in the injured peripheral nerve^41^. The extent of S-100 stainable cells i.e. presence of Schwann cells, was similar in the PLLA nerve guides the autologous nerve grafts and the hollow tubes. Though Schwann cells migrate or proliferate into a nerve guide, or from other devices, such as a nerve allograft, from both the proximal and distal nerve ends, there were more S-100 stainable cells in the PCL nerve guide than in the autograft at 8 mm. This indicates that Schwann cells might respond and migrate/proliferate differently into a nerve guide from the proximal and the distal ends.

To our knowledge, this is also the first report of an *in vivo* study of a porous multi-channeled, electrospun nanofiber nerve guide endowed with supporting cells. Upon application of the cell suspension, the solution appeared to be easily injected and absorbed by the nerve guide. SVF-cells were spread throughout the guides as shown by LipidTOX Red staining. Some of those cells accumulated at the distal end, probably due to gravity as the SVF cell suspension was injected with the nerve guide vertically tilted^42^.

A tubular biograft made from electrospun PCL/gelatin nanofibrous mats has been examined in conjunction to cell therapy with stem cells from human exfoliated deciduous tooth (SHED), to bridge 10 mm sciatic nerve gap in *in vivo* rat models^43^. They report that SHED seeded on nanofibrous nerve guides could survive and promote axonal regeneration in rat sciatic nerves after an observation period of 16 weeks. They did not, however, use micro-channels with their possible mechanical restraint.

In our PLLA fiber nerve guide, cell therapy with SVF-cells did not significantly change the axonal outgrowth (neurofilament staining) compared to nerve guide without cells. There was an increased inflammatory response in the cell therapy group, with more ED1 stainable cells both at the site of lesion and 5 mm distally compared to the group reconstructed with the nerve guide alone, possibly due to cell debris. Further, the extent of Schwann cells (i.e. S-100 stainable cells) was inferior in the PLLA nerve guides seeded with cells. Our interpretation is that the transplanted SVF-cells applied a topographical hindrance. The discrepancy between effects on Schwann cells and axonal outgrowth indicates that the transplanted SVF-cells affected cell migration and/or proliferation rather than axonal regeneration. At all rates, this suggests that such SVF-cells after transplantation are capable of influencing complex biological phenomena in a neurological setting. In combination with the low risk for immunological reactions this should place SVF-cells amongst the interesting possibilities in coming therapeutic initiatives.

Notably, 9/20 PLLA nerve guides in the cell therapy group, which completed the study, were grossly encapsulated at harvest, and appeared to be infected or inflamed. Some sciatic nerves in this group were macroscopically bridged, and some were unbridged. As antibiotics were not given, this reaction may represent a locally controlled implant infection. It may also be an adverse effect similar to the severe inflammatory response, including massive lymphocyte infiltration, after transplantation of SVF-cells, which has been reported in another non-neural setting^44^.

Surgically assisted therapeutic cell transplantation is an event that changes the biological situation in the regenerating peripheral nerve, adding a new cellular component to the microenvironment. Therefore, in order to optimize axonal regeneration, and create a suitable environment for interaction of transplanted cells, while preserving the needs of Schwann cells, the features of the cell delivery vehicle needs further optimization. An increased porosity of the shell, providing opportunity for macrophage invasion, would be preferable if the cell therapy is to be used. The origin of the transplanted cell population could possibly also influence the environmental needs of the ultimate nerve guide.

We conclude that electrospinning can be used to create a geometrical environment inspired by nature’s own design to support peripheral nerve regeneration, perhaps even across distances where hollow tubes cannot be used. In this context, PCL should be avoided. By contrast, PLLA appears to be well tolerated. Our solution of using an external shell for stability is promising for this model, although this needs more development. The demands on multi-channeled nerve scaffolds acting as cell delivery vehicles of SVF *in vivo* are unknown.

## Methods

### Fabrication of Electrospun Nerve Guides

#### Pcl nerve guides

A custom-made needle based electrospinning set up was used for all sample preparation. In short: a syringe with a flat tip needle 21 G was driven by a syringe pump (Dual pump, World Precision Instruments). Two high voltage supplies with common ground was used with the needle connected to the positive electrode, and the collector to the negative, the latter was not critical but helped concentrating the fiber jet around the area of interest; hence reducing the spinning and fabrication time. The voltage was +20 kV (needle) and −2 kV (target), polymer feed rate 2 ml/hr and needle-target distance 25 cm. PCL (Sigma Aldrich, St. Louis, MO, USA) was dissolved in acetone (Kebo, Stockholm, Sweden) to a 14% (w/w) solution. The nerve guides were made using two different collectors in sequence. First, a U-shaped 8 × 20 cm aluminum target was used for aligned fibers. Two plastic combs (1 tooth/mm) were placed on the long edges of the U-plate and served as holders for the channel templates, i.e. 30 threads of 6-0 suture (Teleex, Wayne, PA, USA) mounted between the combs, during the first fabrication step where 0.45 ml PCL was electrospun. Then the whole target was turned 180° and the electrospinning was repeated. By this set up the channel templates could be covered in aligned fibers. Secondly, after careful removal of unwanted fibers between each suture, more mechanically stable random fibers were deposited (0.40 ml PCL) on both sides by changing the U-shaped aluminum collector, to a standard plate-collector. Using scissors, the fiber mat was trimmed close to the edges and the mat was rolled loosely into guides with a diameter of 3–3.5 mm and secured by sutures. The templates were carefully removed using tweezers, which was facilitated by heating the guides gently in oven for two hours at 45 °C. The guides were then soaked in water and frozen at −20 °C to increase their rigidity before subdivide the guide into 10 mm segments. The two segments from the middle of each guide were used for further experiments. Due to the fragility and to make further handling during surgery easier, they were put in 14 mm long silicone tubes (VWR, Radnor, PA, USA) with an inner diameter of 3.5 mm. The final guides consisted of rolled fiber mats, in which up to 30 intact channels with aligned fiber walls could be found. Prior to implantation, guides were sterilized 3 × 10 minutes in 70% ethanol and stored in sterile PBS.

#### PLLA nerve guides

PLLA (Goodfellow, Huntingdon, UK) was dissolved in chloroform to produce a 14 w/v% solution. The electrospinning procedure was done as above, with minor adjustments. The sutures used were 6-0 Ethilon, needle-target distance 20 cm; flow rate 1.5 ml/h with 2 × 300 µl of PLLA solution for aligned fibers; while the flow rate was increased to 2 ml/h and 200 µl of PLLA solution for random fibers on each side of the sutures. Due to mechanical properties, PLLA fiber mats could be rolled up more tightly and were put into 14 mm long silicone tubes with inner diameter 3 mm. As above, the tube-guide complex, as well as empty control tubes, was finally sterilized in ethanol and stored in PBS.

### Nerve Guide Characterization

The guides for characterization were soaked in water, frozen and cut in thin transverse (1–2 mm) slices using a scalpel. Sections, were air dried before sputtered with 12 nm platinum using a JFC-1300 sputterer (Anatech Ltd., Battle Creek, MI, USA) and then imaged with a JSM-5600 field emission scanning electron microscope (JEOL Ltd., Tokyo, Japan). Image analysis was performed using ImageJ (https://imagej.nih.gov/ij/index.html). Three cross-sections with minimal cutting artifacts were selected for analysis, and the number of intact channels remaining after the fabrication were counted. A channel was considered to still be intact if its cross-sectional area was 10,000 µm^2^ or higher. The porosity was approximated by calculating the area of unoccupied space as a percentage of the total guide area in randomly selected sections. The contribution to the porosity from the channels only was calculated in order to determine how much of the free space that was endowed with aligned fibers by manually tracing the edges of the tube or fiber layers in ImageJ (Fig. 1B).

### *In vivo* Experiments

The animal ethics committee in Malmö/Lund (Lunds tingsrätt, Box 75, 221 00 Lund, Sweden) approved all animal experiments, and the experiments were carried out in accordance with the relevant guidelines and regulations. The animals were kept in cages with a 12 h light/dark cycle. Food and water were available *ad libitum*. Female Wistar rats (200–250 g) were bred by Taconic (Denmark). A total of 106 rats were used, and randomly divided into groups as indicated in Fig. 2. The animals were anesthetized by intraperitoneal ketamine (75 mg/kg) and xylazine (10 mg/kg), after which the sciatic nerve was exposed and 10 mm was resected at the thigh level. The 10 mm defect was immediately bridged by a silicone tube with nerve guide; with or without addition of cells (see below); or bridged by a hollow tube; or bridged by an autologous nerve graft, that consisted of the transected 10 mm nerve segment in a reversed orientation.

For the group receiving cell therapy, the groin fat flat was exposed and ~0.7 g of adipose tissue was resected, after which the wound was closed with staples. A stromal cell suspension was created according to a protocol modified from Suganuma^7^. Briefly, the adipose issue was cut into smaller pieces and added to a 0.012% (w/w) collagenase in PBS solution. After 45 minutes of incubation in a 37◦C water bath, with stirring every 15 minutes, the solution was passed through a 100 µm filter into Dulbecco’s Modified Eagle’s Medium (DMEM). The solution was centrifuged at 1300 rpm for 5 min and the supernatant was discarded before the pellet was re-suspended in DMEM and centrifuged again. After discarding the supernatant, the pellet was then re-suspended in PBS. Using a 50 µl pipette, the cell suspension was injected in the lumen of the tube with nerve guide, after attaching it the sciatic nerve distally. With careful handling, especially avoiding tilting the tube and possibly draining it of the cell suspension, the proximal nerve end was then sutured into the nerve guide. The nerve ends were approximated into the tube by end-to-tube suturing with two 9-0 Ethilon sutures, or for autologous nerve graft by three end-to-graft epineurial 9-0 Ethilon sutures. All surgeries were performed by a single surgeon (HF). The animals were given Temgesic for post-operative analgesia, and were allowed to wake up in a heated cage filled with an especially soft filling material to avoid wound development caused by drop foot of the operated limb. After 28 days, the animals were sacrificed with an overdose of pentobarbital and the heart was cut. The sciatic nerves were harvested and trimmed from excess fat and scar tissue.

### Macroscopic Evaluation

The integrity of the nerve conduit, the continuity of regenerated tissue, and any signs of infection were observed at the time of harvest.

### Tissue Preparation

PCL study denotes the part of the study in which PCL guides were used, while PLLA study denotes the part of the study where PLLA guides were used, even though such guides where not used when it came to the respective autologous nerve graft and hollow tube groups.

#### PCL study

Sciatic nerves were fixated in Stefanini solution (4% paraformaldehyde, 1.9% picric acid in 0.1 M phosphate buffer (PBS) pH 7.2) over night; washed trice in 1xPBS; and incubated in 20% sucrose solution overnight for cryoprotection. After storage at −20 °C, samples were embedded in Tissue-Tek O.C.T. (Histolab products AB, Göteborg, Sweden) and a cryotome (Leica Microsystems, Germany) was used to collect transversal sections with a thickness of 20 µm from each sample, gathered proximally; at 2, 5 and 8 mm into the graft; and distally. Sections were collected on glass slides and stored at −20 °C.

#### PLLA study

Sciatic nerves were fixated and cryoprotected as above, embedded in Yazulla medium (30% egg albumin, 3% gelatin in distilled water) and stored at −80 °C. Longitudinal sections were cut with 12 µm thickness using a cryotome (Microm HM560), collected and stored as above.

### Antibodies

#### PCL study

We used a rabbit anti-NF200 (1:800, Sigma Aldrich); goat anti-S-100 (1:100; Santa Cruz Biotechnology, CA, USA); and mouse anti-ED1 14 (1:400; AbD Serotec, Oxford, Great Britain), 1:400, for visualization of neurofilaments, Schwann cells and macrophages, respectively. Primary antibodies were visualized with appropriate Alexa Fluor conjugated antibodies (1:400, Molecular Probes, Eugene, OR, USA). We used the antibody dilution buffer 0.25% Triton-X, 5% fetal serum albumin in 1x phosphate-buffered saline (PBS) pH 7.2. Sections were incubated with 1 g/ml Hoechst 33342 (Sigma Aldrich) in PBS to visualize the cell nuclei; mounted with GlycerGel mounting medium (DakoCytomation, Glostrup, Denmark) and cover slipped.

#### PLLA study

For the PLLA nerve study, we used a monoclonal mouse anti-human neurofilament protein (1:80, DakoCytomation, Glostrup, Denmark); mouse monoclonal anti-ED1 IgG (1:100, sc-59103, Santa Cruz Biotechnology); and mouse monoclonal anti-S-100 IgG1 (1:300, sc-58839, Santa Cruz Biotechnology). Primary antibodies were visualized with appropriate Alexa Fluor conjugated antibodies (1:500, Invitrogen, Lidingö, Sweden). We used the antibody dilution buffer 0.1% Triton-X, 0.25% bovine serum albumin in 1x PBS pH 7.2. Samples were mounted with Vectashield mounting medium with DAPI (Vectashield, Vector Laboratories, Inc. Burlingame, CA 94010, USA) to visualize the cell nuclei, and cover slipped.

### Localization of Transplanted SVF cells - LipidTOX Red Staining

SVF contains a variety of cells with different markers. To determine the presence or absence of adipocytes derived from the SVF cell population in transverse sections four weeks after implantation, some slides from the PLLA nerve guide experiments were stained with LipidTOX Red which stains neutral lipid droplets. We used LipidTOX (Invitrogen, 1:200) according to the manufacturer’s instructions, using 30 min incubation time. Samples were mounted as above. Digital photos were obtained, and imported to ImageJ. Presence of LipidTOX Red stainable cells was observed, but was not quantified.

### Microscopy and Immunofluorescence

#### PCL study

For characterization and immunofluorescence, we used an Olympus U-RFL-T (LRI instrument AB) equipped with Nikon DN-Ri1 and NIS Elements software.

#### PLLA study

We used an Eclipse (Nikon, Tokyo, Japan) equipped with Digital Sight (Nikon, Tokyo, Japan).

### Neurofilament Staining and Image Analysis

#### PCL study

The material characteristics permitted transverse sections only. Neurofilament protein staining was used to visualize axonal regeneration and the front of axons. Randomly selected sciatic nerve sections were stained with anti-NF200 antibody and visualized by secondary antibodies carrying an Alexa Fluor tag (a fluorescent dye). Digital images were analyzed in ImageJ. Systematically randomly sampled sections from each specimen were manually counted, and the average of three observations was retained for further analysis. Proximal and distal to the nerve injury (where the samples were more uniform), and at in the hollow tube (where few sections were available), only one observation per specimen was made.

#### PLLA study

Sciatic nerve sections were stained with anti-neurofilament antibody and visualized by secondary antibodies carrying an Alexa Fluor tag. Blind-coded sections were photomicrographed using the fluorescence microscope and camera above. The length of axonal outgrowth was measured on digital photographs using ImageJ. The longest distance between the suture line and the front of neurofilament-stainable axons (>3 continuously growing stainable axons) was retained for measurements of length in two ways in each individual (maximum observed length, and average neurofilament length from three observations when reconstructed with a nerve guide). For autologous nerve graft and hollow tube, where regeneration was more uniform in space, the analyzes were performed in one systematically randomly sampled section from each specimen. For nanofiber nerve guide groups, with and without cell therapy, we analyzed three systematically randomly sampled sections from each specimen. In the group treated with the hollow tube, the regenerated matrix 28 days after implantation was so thin that it was difficult to capture sections in the cryotome containing the full length of the matrix. For this group, observed presence of neurofilament stainable filaments at the distal nerve lesion was assumed to imply complete bridging of the 10 mm nerve defect.

### S-100 and ED1 Staining, and Total Cell Count

#### PCL study

The distribution of S-100 stainable cells, as a marker for Schwann cells, was investigated. Schwann cells are believed to be most important cell type to enable and support regeneration in the injured peripheral nerve^41^; while activated macrophages – detected by the pan-macrophage marker ED1 – are believed to be of importance in the degeneration process to clear debris and create a permissible microenvironment^45^. Transverse sections, taken proximal of the nerve lesion; at 2, 5 and 8 mm inside the tube; and distal to the lesion were analyzed for S-100 and ED1 stainable structures. For total cell count, digital images of transverse sections stained with Hoechst 33342 (an intercalating agent, binding DNA in the cell nuclei). Systematically randomly sampled sections from each specimen were manually counted, and the average of three observations, if possible, was retained for further analysis. Proximal and distal to the nerve guide (where the samples were more uniform), and in the hollow tube (where few sections were available), only one observation per specimen was made at each distance.

#### PLLA study

Sciatic nerve sections were stained with anti-S-100 antibody, visualized by secondary antibodies carrying an Alexa Fluor tag and analyzed on digital photographs using ImageJ. The longest distance between the suture line and the front of S-100 stainable cells (>3 continuously growing cell extensions) was retained for measurements of length in each individual. One randomly selected observation per specimen was analyzed. Quantification of ED1 stainable macrophages, as a marker for activation of inflammatory response with invading macrophages^45^, was performed by manual cell counting on digital images of longitudinal sections from five systematic randomly sampled regions of interest of 10,000 µm^2^: near the proximal site of lesion (at the suture line for autologous nerve graft, and at the proximal border of the nerve guide, respectively), and 5 mm distally.

### Statistics

The analyzes were performed with the IBM SPSS Statistics 22 software. The non-parametric method Kruskal-Wallis (KW) was used to detect differences between groups, with the post hoc Mann-Whitney (MW) test to investigate differences between treatment groups at specific locations. The significance level was set to 0.05.

## Electronic supplementary material

Supplementary Table S1

## Data Availability

The datasets generated during and analyzed during the current study are available from the corresponding author on reasonable request.
