# Author Correction: Electrospun nerve guide conduits have the potential to bridge peripheral nerve injuries in vivo

**DOI:** 10.1038/s41598-019-44658-6

**Published:** 2019-07-05

**Authors:** Hanna K. Frost, Tomas Andersson, Sebastian Johansson, U. Englund-Johansson, Per Ekström, Lars B. Dahlin, Fredrik Johansson

**Affiliations:** 1 Department of Translational Medicine – Hand Surgery, Lund University, SE-205 02 Malmö, Sweden; 2 Department of Hand Surgery, Skåne University Hospital, SE-205 02 Malmö, Sweden; 3 Department of Biology, Lund University, SE-223 62 Lund, Sweden; 4 Department of Clinical Sciences in Lund - Ophtalmology, Lund University, SE-211 84 Lund, Sweden

Correction to: *Scientific Reports* 10.1038/s41598-018-34699-8, published online 13 November 2018

In this Article, the legend of Figure 1 is incorrect. In addition, panels (C) and (D) of the figure were published by the Authors previously and the Authors neglected to cite this previous paper, which is included below as Reference 1. As a result, the legend of Figure 1,

“PLC nerve guide characteristics. (A) SEM images of the PCL guide with more defined number of micro channels; (B) individual channel dimensions traced in ImageJ to calculate the porosity; (C) randomly oriented PLLA fibers; and (D) aligned PLLA fibers, indicating the porosity of the scaffold, and (E) the Fourier spectra of the random and aligned fibers. (F) SEM image of the PLLA nerve guide, where the pores appear to be less well defined than in the PCL guide; (G) the PCL nerve guide inserted in its silicone shell; (H) explanted nerve guide integrated in the regenerating sciatic nerve; (I) hollow tube bridged by a thin silicone matrix; (J) the regenerated matrix measured to the length of the sciatic nerve defect of 10 mm”.

should read:

“PLC nerve guide characteristics. (A) SEM images of the PCL guide with more defined number of micro channels; (B) individual channel dimensions traced in ImageJ to calculate the porosity; (C) randomly oriented PCL fibers^1^; and (D) aligned PCL fibers^1^, indicating the porosity of the scaffold, and (E) the Fourier spectra of the random and aligned fibers. (F) SEM image of the PLLA nerve guide, where the pores appear to be less well defined than in the PCL guide; (G) the PCL nerve guide inserted in its silicone shell; (H) explanted nerve guide integrated in the regenerating sciatic nerve; (I) hollow tube bridged by a thin silicone matrix; (J) the regenerated matrix measured to the length of the sciatic nerve defect of 10 mm”.

## References

[1] Zalis MC, Johansson S, Johansson F, Johansson UE (2016). Exploration of physical and chemical cues on retinal cell fate. Molecular and cellular neurosciences.

