# Supplementary material for: Electrospun nerve guide conduits have the potential to bridge peripheral nerve injuries in vivo
Source: Sci Rep. 2018 Nov 13;8:16716. doi: 10.1038/s41598-018-34699-8 (PMC6233209; doi:10.1038/s41598-018-34699-8)
Supplement: Supplementary file 1 — Supplementary Table S1 [file 41598_2018_34699_MOESM1_ESM.pdf]

Electrospun nerve guide conduits have the potential to bridge peripheral nerve injuries in vivo

FROST Hanna K*[1,2], ANDERSSON Tomas[3], JOHANSSON Sebastian[3], ENGLUND –JOHANSSON U[4], EKSTRÖM[4] Per, DAHLIN Lars B[1,2], JOHANSSON Fredrik[3].

[1]Department of Translational Medicine – Hand Surgery, Lund University, SE-205 02 Malmö, Sweden

[2] Department of Hand Surgery, Skåne University Hospital, SE-205 02 Malmö, Sweden

[3] Department of Biology, Lund University, SE-223 62 Lund, Sweden

[4] Department of Clinical Sciences, Lund - Ophthalmology, Lund University, SE-211 84, Sweden

*Correspondence to Hanna.Frost@med.lu.se

E-mail addresses: Hanna.Frost@med.lu.se (H. Frost), seb.m.johansson@gmail.com (S. Johansson), tomas.k.andersson@gmail.com (T. Andersson), Ulrica.Englund_johansson@med.lu.se (U Englund-Johansson), Per.Ekstrom@med.lu.se (P. Ekström), Lars.Dahlin@med.lu.se (LB. Dahlin), Per_Fredrik.Johansson@biol.lu.se (F. Johansson)

Table S1. Nerve regeneration after nerve injury and reconstruction with PCL nerve guide

	Autograft	Hollow tube	PCL nerve guide	PCL nerve guide+cells	p-values (KW)
**NF per mm^2**	n=5	n=2	n=3	n=2	
prox	32235 (30146, 34026)	3326 (2875, 3776)	3326 (2875, 3776)		p=0.153
2mm	22037 (12436, 26962)	2050 (1047, 5106)	2050 (1047, 5106)	2234 (972, 3496)	p=0.075
5mm	19785 (16565, 23005)	664 (611, 718)	664 (611, 718)	1642 (972, 2313)	p=0.112
8mm	17088 (15123, 22982)	991 (761, 1221)	991 (761, 1221)	870[‡]	p=0.081
dist	3507 (0, 10745)		746 (149, 1343)	448[‡]	p=0.570
**Hoechst 33342 per mm^2**	n=5	n=2	n=4	n=2	
prox	12237 (7760, 13133)	15670[‡]	12834 (12685, 14774)	12536[‡]	p=0.130
2mm	21639 (8506, 23380)	27061[‡]	14749 (10447, 20296)	16167 (13730, 18605)	p=0.307
5mm	19351 (15471, 27310)	66012 (25669, 106355)	14177 (10546, 14426)[a]	14600 (11939, 17262)	**p=0.040**
8mm	20097 (17311, 21440)	24475[‡]	10049 (5820, 12188)[a]	15446 (15322, 15570)	**p=0.020**
dist	16267 (14177, 19550)	13730[‡]	15297 (12237, 18804)	15670 (14476, 16864)	p=0.517
**ED1 per mm^2**	n=5	n=3	n=3	n=2	
prox	454 (48, 883)	167 [‡]	1122 (24, 1170)	263[‡]	p=0.963
2mm	1703 (24, 2260)	2260 (1242, 3852)	485 (40, 1504)	1839 (1321, 2356)	p=0.280
5mm	1031 (637, 1425)	1655 (1464, 5587)	527 (334, 2650)	1327 (1186, 1468)	p=0.259
8mm	1401 (1337, 1854)	3868 (1051, 6685)	850 (167, 1743)	1166 (883, 1449)	p=0.508
dist	1647 (48, 2244)	4775 (4202, 5348)	728 (454, 1289)	1391 (1170, 1612)	p=0.095
**S100 per mm^2**	n=5	n=2	n=4	n=2	
prox	96 (0, 167)	669[‡]	24 (0, 167)	167[‡]	p=0.435
2mm	72 (0, 111)	905 (414, 1396)	621 (493, 836)	489 (119, 860)	p=0.059
5mm	48 (48, 127)	658 (350, 966)	1019 (0, 1592)	561 (478, 645)	p=0.239
8mm	135 (0, 382)	1487[‡]	852 (382, 1098)[a]	1242[‡]	**p=0.049**
dist	60 (0, 143)	3629[‡]	669[‡]	609 (454, 764)	p=0.075

Table S1. Nerve regeneration after nerve injury and reconstruction with PCL nerve guide.

Data from the initial PCL-tube study analyzed on photograps of transverse sections at different tube levels. Due to a high degree of automutilation and processing difficulties, groups are small. Values are expressed as median (min, max), and are based on the average of three observations per specimen, except for proximal and distal to the nerve injury, where the nerve was uniform. For the hollow tube, values are based on a single observation per specimen due to a shortage of sections and a uniform distribution of cells. KW= Kruskal-Wallis. Statistical significance ($p < 0.05$) based on the Mann-Whitney U-test is indicated as: [a] sign. difference from group 1 (autologous nerve graft). [†] denotes that observation is based on a single specimen.
